# Estimation of minimally important differences in EQ-5D utility and VAS scores in cancer

**DOI:** 10.1186/1477-7525-5-70

**Published:** 2007-12-21

**Authors:** A Simon Pickard, Maureen P Neary, David Cella

**Affiliations:** 1 Center for Pharmacoeconomic Research, Department of Pharmacy Practice, College of Pharmacy, University of Illinois at Chicago, Chicago, USA; 2 Global Health Outcomes, GlaxoSmithKline, Collegeville, Pennsylvania, USA; 3 Center for Outcomes Research and Education, Evanston Healthcare and Feinberg School of Medicine, Northwestern University, Chicago, USA

## Abstract

**Background:**

Understanding what constitutes an important difference on a HRQL measure is critical to its interpretation. The aim of this study was to provide a range of estimates of minimally important differences (MIDs) in EQ-5D scores in cancer and to determine if estimates are comparable in lung cancer.

**Methods:**

A retrospective analysis was conducted on cross-sectional data collected from 534 cancer patients, 50 of whom were lung cancer patients. A range of minimally important differences (MIDs) in EQ-5D index-based utility (UK and US) scores and VAS scores were estimated using both anchor-based and distribution-based (1/2 standard deviation and standard error of the measure) approaches. Groups were anchored using Eastern Cooperative Oncology Group performance status (PS) ratings and FACT-G total score-based quintiles.

**Results:**

For UK-utility scores, MID estimates based on PS ranged from 0.10 to 0.12 both for all cancers and for lung cancer subgroup. Using FACT-G quintiles, MIDs were 0.09 to 0.10 for all cancers, and 0.07 to 0.08 for lung cancer. For US-utility scores, MIDs ranged from 0.07 to 0.09 grouped by PS for all cancers and for lung cancer; when based on FACT-G quintiles, MIDs were 0.06 to 0.07 in all cancers and 0.05 to 0.06 in lung cancer. MIDs for VAS scores were similar for lung and all cancers, ranging from 8 to 12 (PS) and 7 to 10 (FACT-G quintiles).

**Discussion:**

Important differences in EQ-5D utility and VAS scores were similar for all cancers and lung cancer, with the lower end of the range of estimates closer to the MID, i.e. 0.08 for UK-index scores, 0.06 for US-index scores, and 0.07 for VAS scores.

## Background

It is common, if not usual practice, to include health-related quality of life (HRQL) measures in clinical trials in oncology. To justify the cost of new cancer drugs, decision-makers need to determine not only whether a drug has a statistically significant impact on survival and/or HRQL, but they also need to evaluate whether the improvement is meaningful. This is particularly important in lung cancer, where aggressive new therapies are being brought to market. In addition to the use of cancer-specific measures such as European Organization for Research and Treatment of Cancer-QLQ-C30 (EORTC QLQ-C30) [1,2]and the Functional Assessment of Chronic Illness Therapy (FACIT) measurement system [3], clinical trials in oncology are increasingly incorporating generic preference-based measures such as EQ-5D. EQ-5D is an indirect measure of utility for health that generates an index-based summary score based upon societal preference weights [4]. Utility scores enable comparisons of burden of disease across conditions and the calculation of quality-adjusted life-years (QALYs), an outcome used to compare the cost effectiveness of health care technologies.

A major challenge in HRQL measurement is the interpretation of scores, particularly with respect to defining what constitutes a minimally important difference (MID). The MID has been defined as the smallest change in a PRO measure that is perceived by patients as beneficial or that would result in a change in treatment [5]. Approaches to estimation of MIDs have been classified as either distribution-based or anchor-based [6]. Anchor-based approaches compare changes seen in an individual's HRQL to an external criterion, such as a clinical measure or using a patient rated global change question. Problematically, no single anchor represents a gold standard and no approach is ideal. Norman et al (1997) found that retrospective global ratings of change have questionable ability to yield information of treatment effects [7]. Alternatively, distribution-based approaches rely on the distribution of scores and are computed using variations on effect size [8]. The main disadvantage to distribution-based techniques is that they do not provide insight into the importance of the difference [9]. Often both approaches are combined, with anchored-based HRQL changes initially framed in terms of the individual are then further analyzed as a group using distribution-based methods [10-15].

While MIDs have been estimated for EQ-5D index-based scores for some conditions [16], empiric work has not been performed in cancer. Additionally, it is not clear if lung cancer has a different range of MID estimates. Thus, the aim of this study was to provide a range of estimates for meaningful difference in EQ-5D scores in cancer and to determine if MIDs for lung cancer are different from all cancers.

## Methods

### Study design

A retrospective analysis was conducted on cross-sectional data collected from 534 cancer patients with eleven types of cancer who participated in a validation study of cancer symptoms scales [17]. Participants had advanced (stage 3 or 4) cancer of the bladder, brain, breast (females patients only), colon/rectum, head/neck, liver/pancreas, kidney, lung, lymphoma, ovary (females patients only), and prostate (males patients only). All patients had received at least 2 cycles of chemotherapy, or if chemotherapy was non-cyclical, had been receiving it for at least 1 month. Efforts were made to recruit 50 patients for each type of cancer, with approximately equal proportions of male and female patients for the non-gender specific types of neoplasm. This dataset included 50 patients lung cancer patients, and between 50 and 52 patients with all other types of cancer except bladder cancer (n = 31).

The patients were recruited from six sites within the National Cancer Coalition Network (NCCN) and the Cancer Health Alliance of Metropolitan Chicago (CHAMC). The NCCN is a not for profit, tax-exempt corporation that is an alliance of National Cancer Institute (NCI) approved comprehensive cancer centers. The CHAMC organizations provide social, emotional and informational support services to cancer patients free of charge. These organizations are not affiliated with a medical center or university, and each CHAMC agency serves different geographical and socio-demographic cancer patient populations. All patients who completed the questionnaires consented to participate in the study. Institutional review board approval was obtained for secondary data analysis (University of Illinois at Chicago research protocol #2006-0891).

### Measures

Patients completed several questionnaires, including the EQ-5D and the Functional Assessment of Cancer Therapy (FACT). The EQ-5D descriptive system consists of 5 dimensions: Mobility, Self-Care, Usual Activities, Pain/Discomfort, and Anxiety/Depression, each with 3 levels (e.g. no problems, moderate problems, extreme problems) [18]. Index-based summary scores were calculated based on 2 different algorithms using societal preference developed from general population-based valuation studies in the United Kingdom [19] and the USA [20]. The index-based score is typically interpreted along a continuum where 1 represents best possible health and 0 represents dead, with some health states being worse than dead (<0). In addition to the self-classifier, respondents rate their health today using a 20 centimeter visual analogue scale (VAS) that ranges from 0 (worst imaginable health state) to 100 (best imaginable health state).

Participants also completed the Functional Assessment of Cancer Therapy (FACT) quality of life questionnaire using a version specific to their tumor type. The general subscales common to all versions (FACT-G) include physical well-being (PWB), social/family well-being (SFWB), emotional well-being (EWB), and functional well-being (FWB). The FACT-G total score (FACT-G Total) is based on 26 summed items (responses 0 to 4) from the PWB (7 items), FWB (7 items), SFWB (6 items), and EWB (6 items). Higher scores represent better quality of life.

Performance status was evaluated using the Eastern Cancer Oncology Group (ECOG) classification system [21]. ECOG grades range from 0, which is fully active, to 4, completely disabled, and 5 is dead. ECOG grades are used by physicians and researchers to assess progression of disease, impact of the disease on daily activities, and to guide appropriate treatment and prognosis.

### Analysis

Both anchor-based and distribution-based approaches were used to estimate MIDs for the EQ-5D in the overall cancer cohort, and in the subgroup of lung cancer patients, when possible. Distribution-based criteria included: 1/2 standard deviation (SD) and the standard error of the measure (SEM) [22]. For consistency with past studies exploring MIDs, 1/3 SD was also reported, but it was not included in the summarized range of MIDs as there is less evidence to support that 1/3 SD represents an important difference. The SEM is calculated as $\sigma_{\text{x}}*\sqrt{1-r_{\text{x}}}$ where r is reliability of the measure. It is debatable which type of reliability, internal consistency or test-retest (TRT) reliability, is most appropriate. Very limited evidence of TRT reliability is available on the EQ-5D in cancer [4]. Because the EQ-5D has single item dimensions, internal consistency reliability does not apply to each dimension. Although HRQL is considered a multi-dimensional construct, the aggregation of dimensional responses to create a single summary score is an implicit endorsement of HRQL as an overarching construct. However, item response theory-based analysis of the dimensional structure of the EQ-5D has indicated that the anxiety/depression dimension taps into a construct distinct from the other 4 items [23]. Calculation of internal consistency reliability using Cronbach's alpha was 0.68, regardless of whether or not anxiety/depression was included. Thus, for the purposes of our analysis, a reliability of 0.68 was used in the calculation of the SEM.

Anchors can be constructed using clinically-based criteria, such as response to treatment, or more subjective criteria, e.g. health status. We used ECOG grades, assessed by physician, to group patients into categories of performance status, and determined mean difference scores between ECOG grades. Distribution-based criteria were then applied to the statistics associated with each anchor-based category. A second anchor-based approach used FACT-G scores. The cohort was stratified into quintiles based on FACT-G summary scores. Grouping the cohort into quintiles approximated an appropriate threshold for stratifying patients based on MID estimates for the FACT-G, have been identified as close to 6 in previous studies: 6–7 in hepatobiliary carcinoma [13], and 5–6 in breast cancer [10]. Final results were summarized as a range of MID estimates and as an average MID across categories, weighted by the sample size within each category.

## Results

Similar demographic characteristics were observed in the overall cancer sample and the lung cancer subgroup (Table 1). A wide range of scores were observed in the overall cancer cohort, with UK-based scores ranging from worse than dead (-0.14) to full health (1.0). A smaller range was observed for the US-based scores (0.21 to 1.0). Compared to the mean (SD) scores for the overall cohort [UK 0.72 (SD 0.22); US 0.78 (SD 0.15)], the subgroup with lung cancer had lower mean utility scores but similar dispersion around the mean [UK 0.67 (SD 0.22); US 0.74 (SD 0.16)] (Table 2). Mean VAS scores for the lung cancer subgroup [68 (SD 18)] were the same as for the overall cancer cohort [68 (SD 20)].

**Table 1:** Patients characteristics, all cancers and lung cancer subgroup

	**All cancers (n = 534)**	**Lung cancer (n = 50)**
**Characteristic**		

Age (mean, SD)	59 (12)	62 (10)
Gender – female (n, %)	258 (48%)	26 (59%)
Race (n)		
White	474 (89%)	40 (91%)
Black	44 (8%)	3 (7%)
Other	15 (3%)	1 (2%)
Of Spanish/Hispanic/Latino ancestry	16 (3%)	0 (0%)
ECOG level		
0	132 (25%)	8 (18%)
1	263 (49%)	30 (68%)
2	76 (14%)	5 (11%)
3	15 (3%)	1 (2%)

ECOG – Eastern Cancer Oncology Group (ranges from grade 0 which is fully active to grade 3, capable of only limited self-care and confined to bed more than 50% of waking hours)

**Table 2:** Patients EQ-5D and FACT-G summary scores, all cancers and lung cancer subgroup

**Cancer Group**	**Score**	**Mean**	**SD**	**Median**	**Min**	**Max**
All (n = 534)	EQ-5D Index US	0.78	0.15	0.81	0.21	1.00
	EQ-5D Index UK	0.72	0.22	0.74	-0.14	1.00
	EQ-5D VAS	68	20	70	0	100
	Fact-G PWB	20	6	21	1	28
	Fact-G SFWB	23	5	24	1	28
	Fact-G EWB	17	3	17	6	24
	Fact-G FWB	16	4	16	4	26
	FACT-G (0 to 108)	79	13	80	36	107
	Total FACT-G (0 to 104)	76	13	77	36	102

Lung (n = 50)	EQ-5D Index US	0.74	0.16	0.77	0.31	1.00
	EQ-5D Index UK	0.67	0.22	0.69	0.08	1.00
	EQ-5D VAS	68	18	73	25	95
	Fact-G PWB	20	5	21	1	28
	Fact-G SFWB	23	4	24	12	28
	Fact-G EWB	17	3	18	10	23
	Fact-G FWB	16	4	16	7	24
	FACT-G (0 to 108)	79	12	80	48	100
	Total FACT-G (0 to 104)	76	12	77	45	99

FACT-G – Functional Assessment of Cancer Therapy General; VAS – Visual Analog Scale; UK – United Kingdom; US – United States; SD – standard deviation; PWB – physical well-being; FWB – functional well-being; SFWB – social/family well-being/EWB – emotional well-being.

For all cancer patients, mean difference scores anchored by ECOG status ranged from 0.09 to 0.16 for UK scores and from 0.07 to 0.11 for US scores (Table 3). Across ECOG-based strata, MIDs based on the SEM and 0.5 SD were similar, ranging from 0.08 to 0.16 for UK scores, and from 0.06 to 0.10 for US scores. For the lung cancer cohort (excluding the single patient with grade 3 PS), mean difference scores between ECOG levels ranged from 0.10 to 0.13 (UK scores), and from 0.07 to 0.09 (US scores). MIDs based on SEM and 0.5 SD ranged from 0.08 to 0.14 (UK scores), and from 0.07 to 0.12 (US scores).

**Table 3:** EQ-5D index-based utility scores by ECOG grade, overall and lung cancer

**Cancer Group**	**ECOG Grade**	**Utility score**	**n**	**Mean**	**SD**	**Med**	**Min**	**Max**	**Mean Diff**	**SEM**	**0.50 SD**	**0.33 SD**
All	0	UK	122	0.85	0.16	0.85	0.16	1.00		0.09	0.08	0.05
		US		0.89	0.11	0.84	0.51	1.00		0.06	0.06	0.04
	1	UK	258	0.73	0.20	0.74	-0.14	1.00	0.13	0.12	0.10	0.07
		US		0.78	0.14	0.81	0.21	1.00	0.10	0.08	0.07	0.05
	2	UK	133	0.63	0.21	0.69	-0.11	1.00	0.09	0.12	0.11	0.07
		US		0.72	0.14	0.77	0.28	1.00	0.07	0.08	0.07	0.05
	3	UK	21	0.48	0.28	0.52	0.02	1.00	0.16	0.16	0.14	0.09
		US		0.61	0.19	0.60	0.26	1.00	0.11	0.11	0.10	0.06

Mean weighted MID		UK	534						0.12	0.11	0.10	0.07
		US							0.09	0.08	0.07	0.05

Lung	0	UK	9	0.78	0.15	0.73	0.62	1.00		0.08	0.07	0.05
		US		0.83	0.11	0.80	0.71	1.00		0.06	0.05	0.04
	1	UK	29	0.68	0.24	0.80	0.08	1.00	0.10	0.14	0.12	0.08
		US		0.74	0.17	0.82	0.31	1.00	0.09	0.10	0.09	0.06
	2	UK	11	0.55	0.18	0.62	0.29	0.76	0.13	0.10	0.09	0.06
		US		0.67	0.12	0.71	0.45	0.83	0.07	0.07	0.06	0.04
	3	UK	1	0.52	--	0.52	0.52	0.52	0.03			
		US		0.60	--	0.60	0.60	0.60	0.07			

Mean Weighted MID		UK	50						0.11	0.12	0.10	0.07
		US							0.09	0.08	0.07	0.05

ECOG – Eastern Cancer Oncology Group (grade ranges from level 0 is fully active to level 3, capable of only limited self-care and confined to bed more than 50% of waking hours); MID – minimally important difference; UK – United Kingdom; US – United States; SEM – standard error of the mean; SD – standard deviation

Average mean estimates of MIDs across FACT-G based quintiles for the overall cancer cohort were 0.09 for UK scores, 0.06 for US scores (Table 4). Using distribution based criteria averaged across quintile-based groups, MIDs for the overall cohort were: SEM_UK _= 0.10, 1/2 SD_UK _= 0.09; SEM_US _= 0.07, 1/2 SD_US _= 0.06. For the lung cancer subgroup, average MIDs between quintiles were 0.10 (UK) and 0.07 (US), with SEM_UK _= 0.09, 1/2 SD_UK _= 0.08; SEM_US _= 0.06, 1/2 SD_US _= 0.06.

**Table 4:** MID estimates for EQ-5D Index-based scores by FACT-G quintile subgroups

**EQ-5D scores**													
**Index Score**	**Cancer Group**	**FACT Quintile**	**FACT mean**	**n**	**Mean**	**SD**	**Med**	**Min**	**Max**	**Mean Diff**	**SEM**	**0.5 SD**	**0.33 SD**

UK	All	1	56.7	103	0.52	0.23	0.62	-0.14	1.00	0.15	0.13	0.12	0.08
		2	68.9	108	0.68	0.17	0.69	0.09	1.00	0.07	0.10	0.08	0.06
		3	76.9	111	0.75	0.19	0.76	0.08	1.00	0.04	0.11	0.09	0.06
		4	83.7	101	0.78	0.17	0.80	0.02	1.00	0.10	0.10	0.09	0.06
		5	92.7	107	0.89	0.14	0.88	0.20	1.00		0.08	0.07	0.05
	
	Mean MID									**0.09**	**0.10**	**0.09**	**0.06**
	
	Lung	1	56.7	7	0.59	0.05	0.62	0.52	0.62	0.03	0.03	0.02	0.02
		2	68.9	11	0.61	0.17	0.66	0.26	0.81	0.18	0.10	0.08	0.06
		3	76.9	6	0.79	0.11	0.80	0.69	1.00	-0.04	0.06	0.06	0.04
		4	83.7	10	0.76	0.20	0.78	0.24	1.00	0.13	0.11	0.10	0.07
		5	92.7	16	0.89	0.13	0.94	0.56	1.00		0.07	0.07	0.04
	
	Mean MID									**0.08**	**0.08**	**0.07**	**0.04**

US	All	1	56.7	103	0.65	0.15	0.71	0.21	1.00	0.10	0.08	0.07	0.05
		2	68.9	108	0.75	0.11	0.77	0.45	1.00	0.05	0.06	0.06	0.04
		3	76.9	111	0.80	0.14	0.82	0.31	1.00	0.03	0.08	0.07	0.05
		4	83.7	101	0.82	0.13	0.83	0.26	1.00	0.08	0.07	0.06	0.04
		5	92.7	107	0.90	0.11	0.86	0.35	1.00		0.06	0.06	0.04
	
	Mean MID									**0.06**	**0.07**	**0.06**	**0.04**
	
	Lung	1	56.7	7	0.68	0.05	0.71	0.60	0.71	0.03	0.03	0.03	0.02
		2	68.9	11	0.71	0.10	0.76	0.52	0.82	0.13	0.06	0.05	0.03
		3	76.9	6	0.84	0.08	0.84	0.77	1.00	-0.04	0.05	0.04	0.03
		4	83.7	10	0.81	0.15	0.84	0.41	1.00	0.10	0.09	0.08	0.05
		5	92.7	16	0.91	0.11	0.93	0.63	1.00		0.06	0.05	0.04
	
	Mean MID									**0.06**	**0.06**	**0.05**	**0.03**

FACT-G – Functional Assessment of Cancer Therapy-General; MID – minimally important difference; UK – United Kingdom; US – United States; SEM – standard error of the mean; SD – standard deviation

MID estimates for EQ-5D VAS scores based on FACT-G score quintiles were the same for both the overall cancer groups and the lung cancer subgroup (Table 5). MIDs for VAS scores ranged from 7 to 10 when MIDs were averaged across the anchor-based categories using FACT-G quintiles. Average mean difference was 7 between quintile categories; 10 according to the SEM; and 9 using 1/2 SD. MIDs for VAS scores tended to be slightly larger using ECOG grade to anchor difference scores compared to FACT-G score based quintiles, ranging from 8 to 11 (all cancers) and 7.5 to 11.5 (lung cancer).

**Table 5:** MID estimates for EQ-5D VAS scores by ECOG grade and FACT-G quintile

Cancer Group	Quintile FACT	FACT mean	n	Mean	SD	Median	Mean Diff	SEM	0.5 SD	0.33 SD
All	1	56.7	102	52	18	50	11	10	9	6
	2	68.9	107	63	18	70	8	10	9	6
	3	76.9	111	71	18	70	5	10	9	6
	4	83.7	101	76	16	80	3	9	8	5
	5	92.7	107	78	19	80		11	9	6

Mean MID							**7**	**10**	**9**	**6**

Lung	1	56.7	7	45	19	50	16	11	9	6
	2	68.9	11	61	18	60	21	10	9	6
	3	76.9	6	82	7	85	-7	4	4	2
	4	83.7	10	75	18	75	0	10	9	6
	5	92.7	16	75	24	80		14	12	8

Mean MID							**7**	**10**	**9**	**6**

	ECOG Grade									

All	0		122	77	19	80	8	11	10	6
	1		258	69	18	70	8	10	9	6
	2		133	61	20	60	3	11	10	7
	3		21	57	23	50		13	12	8

Mean MID							**8**	**11**	**10**	**6**

Lung	0		9	80	11	80	9	6	5	4
	1		29	71	16	75	17	9	8	5
	2		11	54	17	69	24	10	9	6
	3		1	30		30				

Mean MID							**12**	**9**	**8**	**5**

ECOG – Eastern Cancer Oncology Group (grade ranges from level 0 is fully active to level 3, capable of only limited self-care and confined to bed more than 50% of waking hours); MID – minimally important difference; SEM – standard error of the mean; SD – standard deviation

## Discussion

Interpretation of scores is an important issue in the field of HRQL measurement, but there is no consensus on the most appropriate method for assessing the ability of an instrument to capture meaningful differences. In this study, we followed criteria established in previous investigations of MIDs [13-15]. We found that distribution and anchor-based estimates tended to converge, helping to triangulate support for the validity of the range of MID estimates. In addition, the MIDs for overall cancer and lung cancer cohorts were similar.

The issue of what constitutes an MID on a measure of HRQL is part of an ongoing dialogue about issues of interpretation. Developers of HRQL measures have not been forthcoming in the literature in explicitly attempting to establish MIDs. One reason to avoid this is because clinically important differences may vary with the target population. Limitations in the scaling properties of a measure can contribute to inconsistent MID estimates, as they may depend upon where a patient or group falls along the continuum of the measure. Distribution-based approaches for estimating important differences rely on the assumption of normality, and ceiling effects particularly in healthier patient populations produce skewed score distributions. Although ceiling effects have been associated with the use of EQ-5D [24], a ceiling effect was generally not observed in the cancer cohort, and standard deviations were relatively stable across the anchor-based strata.

MID estimates for EQ-5D in this study can be compared to other studies that have examined important differences using EQ-5D. A previous study by Walters and Brazier compared minimally important differences between SF-6D and EQ-5D, and reported a mean MID of 7.4 for the UK-based algorithm [16]. Their estimate was at lower range of MIDs estimated in this study for cancer patients, which may imply that MIDs in cancer are slightly larger than for the conditions investigated, which included leg ulcer, back pain, early rheumatoid arthritis, limb reconstruction, osteoarthritis, irritable bowel syndrome, and chronic obstructive lung disease. An alternative explanation is that the anchors used in this study, particularly ECOG grade, provided benchmarks for meaningful differences that do not necessarily represent a minimally important difference.

MIDs are often estimated using longitudinal datasets, and difference scores based on changes over time were not available in this dataset, which was cross-sectional. However, the MIDs for EQ-5D UK-based utility scores reported using longitudinal data [16] were comparable to the estimates for UK scores generated in this study. Another limitation of our study was that sample size for lung cancer subgroups was small. When further stratified by ECOG grade, sub-sample sizes became extremely small and produced unreliable estimates in the lung cancer subgroup, although the average MID obtained in lung cancer tended to be similar to the overall cancer cohort. It is unclear if MIDs based on patients with advanced cancer in this study generalize to patients with less advanced stages of cancer.

An additional issue for users of EQ-5D is the selection of preference-based algorithm. As observed in this study, MIDs varied with the selection of the algorithm. MIDs for EQ-5D UK index-based utility scores ranged from 0.08 to 0.16 with a mode of 0.10. For US-based scores, a range of 0.06 to 0.12 was reported, with a mode of 0.07. This result was not unexpected, as the US preference-based algorithm produces scores with a smaller range than the UK scores, resulting in smaller difference scores and smaller standard deviations, thus smaller MIDs.

## Conclusion

In summary, important differences in EQ-5D summary scores were similar for all cancers and lung cancer, with the lower bounds likely to represent a closer estimate of true MID, i.e. 0.08 for UK-based scores, 0.06 for US-based scores, and 0.07 for VAS scores. MIDs for EQ-5D UK-based utility scores in cancer were similar to estimated MIDs for other conditions in the published literature. To our knowledge, MIDs for EQ-5D VAS scores and US-based utility scores have not been previously reported. Across the different approaches, MIDs for US-based utility scores were consistently smaller than MIDs for UK-based utility scores.

## Abbreviations

MID – minimally important difference

HRQL – health-related quality of life

FACT-G – Functional Assessment of Cancer Therapy – General

SEM – standard error of the measure

SD – standard deviation

PWB – physical well-being,

SFWB – social/family wellbeing

EWB – emotional well-being

FWB – functional well-being

ECOG – Eastern Cancer Oncology Group

PS – performance status

## Competing interests

A. Simon Pickard is a member of the executive committee of the EuroQol group, a not for profit group that developed and distributes the EQ-5D. David Cella is developer of the FACIT measurement system. Drs. Pickard and Cella have received consulting fees from GlaxoSmithKline, which financed this manuscript including the article-processing charge. They do not have any stocks or shares in an organization that may gain or lose financially from the publication of this manuscript.

## Authors' contributions

ASP, MN and DC were responsible for the conception of the study. ASP and DC acquired the data. ASP performed the data analysis and drafted the manuscript. MN and DC revised it critically for intellectual content, and all authors approved of the final version.
