# Estimation of minimally important differences in EQ-5D utility and VAS scores in cancer

**DOI:** 10.1186/1477-7525-8-4

**Published:** 2010-01-13

**Authors:** A Simon Pickard, Maureen P Neary, David Cella

**Affiliations:** 1 Center for Pharmacoeconomic Research, Department of Pharmacy Practice, College of Pharmacy, University of Illinois at Chicago, Chicago, USA; 2 Global Health Outcomes, GlaxoSmithKline, Collegeville, Pennsylvania, USA; 3 Center for Outcomes Research and Education, Evanston Healthcare and Feinberg School of Medicine, Northwestern University, Chicago, USA

## Correction

We wish to correct a mistake in the abstract and conclusion of our published paper [1]. In the abstract and conclusion, the MID for EQ-VAS score should be reported as 7 rather than 0.07. EQ-VAS scores range from 0 to 100, while EQ-5D index-based scores are anchored by 0 (dead) and 1 (perfect health). The specific wording in the conclusion of the abstract should read "Important differences in EQ-5D utility and VAS scores were similar for all cancers and lung cancer, with the lower end of the range of estimates closer to the MID, i.e. 0.08 for UK-index scores, 0.06 for US-index scores, and 7 for VAS scores.
